# Antigenicity and Diagnostic Potential of Vaccine Candidates in Human Chagas Disease

**DOI:** 10.1371/journal.pntd.0002018

**Published:** 2013-01-17

**Authors:** Shivali Gupta, Xianxiu Wan, Maria P. Zago, Valena C. Martinez Sellers, Trevor S. Silva, Dadjah Assiah, Monisha Dhiman, Sonia Nuñez, John R. Petersen, Juan C. Vázquez-Chagoyán, Jose G. Estrada-Franco, Nisha Jain Garg

**Affiliations:** 1 Department of Microbiology and Immunology, University of Texas Medical Branch, Galveston, Texas, United States of America; 2 Instituto de Patología Experimental, CONICET, Salta, Argentina; 3 Hospital Público de Gestión Descentralizada San Bernardo, Salta, Argentina; 4 Department of Pathology, University of Texas Medical Branch, Galveston, Texas, United States of America; 5 Facultad de Medicina Veterinaria y Zootecnia, Universidad Autónoma de Estado de México, Toluca, México; 6 Faculty of the Center for Tropical Diseases, and the Institute for Human Infections and Immunity, University of Texas Medical Branch, Galveston, Texas, United States of America; Federal University of São Paulo, Brazil

## Abstract

**Background:**

Chagas disease, caused by *Trypanosoma cruzi*, is endemic in Latin America and an emerging infectious disease in the US and Europe. We have shown TcG1, TcG2, and TcG4 antigens elicit protective immunity to *T. cruzi* in mice and dogs. Herein, we investigated antigenicity of the recombinant proteins in humans to determine their potential utility for the development of next generation diagnostics for screening of *T. cruzi* infection and Chagas disease.

**Methods and Results:**

Sera samples from inhabitants of the endemic areas of Argentina-Bolivia and Mexico-Guatemala were analyzed in 1^st^-phase for anti-*T. cruzi* antibody response by traditional serology tests; and in 2^nd^-phase for antibody response to the recombinant antigens (individually or mixed) by an ELISA. We noted similar antibody response to candidate antigens in sera samples from inhabitants of Argentina and Mexico (n = 175). The IgG antibodies to TcG1, TcG2, and TcG4 (individually) and TcG_mix_ were present in 62–71%, 65–78% and 72–82%, and 89–93% of the subjects, respectively, identified to be seropositive by traditional serology. Recombinant TcG1- (93.6%), TcG2- (96%), TcG4- (94.6%) and TcG_mix_- (98%) based ELISA exhibited significantly higher specificity compared to that noted for *T. cruzi* trypomastigote-based ELISA (77.8%) in diagnosing *T. cruzi*-infection and avoiding cross-reactivity to *Leishmania* spp. No significant correlation was noted in the sera levels of antibody response and clinical severity of Chagas disease in seropositive subjects.

**Conclusions:**

Three candidate antigens were recognized by antibody response in chagasic patients from two distinct study sites and expressed in diverse strains of the circulating parasites. A multiplex ELISA detecting antibody response to three antigens was highly sensitive and specific in diagnosing *T. cruzi* infection in humans, suggesting that a diagnostic kit based on TcG1, TcG2 and TcG4 recombinant proteins will be useful in diverse situations.

## Author Summary

Chagas disease is the most common cause of congestive heart failure related deaths among young adults in the endemic areas of South and Central America and Mexico. Diagnosis and treatment of *T. cruzi* infection has remained difficult and challenging after 100 years of its identification. In >95% of human cases, *T. cruzi* infection remains undiagnosed until several years later when chronic evolution of progressive disease results in clinical symptoms associated with cardiac damage. Diagnosis generally depends on the measurement of *T. cruzi*–specific antibodies that can result in false positives. A conclusive diagnosis of *T. cruzi* infection thus often requires multiple serological tests, in combination with epidemiological data and clinical symptoms. In this study, we investigated the antibody response to TcG1, TcG2, and TcG4 in clinically characterized chagasic patients. These antigens were identified as vaccine candidates and shown to elicit protective immunity to *T. cruzi* and Chagas disease in experimental animals. Our data show the serology test developed using the TcG_mix_ (multiplex ELISA) is a significantly better alternative to epimastigote extracts currently used in *T. cruzi* serodiagnosis or the trypomastigote lysate used in this study for comparison purposes.

## Introduction

The protozoan parasite *Trypanosoma cruzi*, transmitted by blood-sucking triatomines, causes Chagas disease, which is a health threat for an estimated 10 million people, living mostly in Latin America. More than 25 million people are at risk of the disease [1]. Increasing travel and immigration has also brought *T. cruzi* infection into non-endemic countries, e.g., the U.S., Spain and Australia, where natural transmission is absent or very low. The congenital and transfusion- or organ transplantation-related transmissions are becoming recognized as significant threats in recent decades [2], [3].

Diagnosis and treatment of *T. cruzi* infection has remained difficult and challenging after 100 years of its identification. This is because the acute infection, in general produces mild clinical symptoms, e.g., fever, dyspnea, local swelling at the site of infection, that are infrequently reported [4]. As a result, acute exposure when detection of blood parasitemia and treatment is possible, remain largely unnoticed. Only those who develop severe acute myocarditis or when an outbreak of *T. cruzi* infection occurs may receive early diagnosis and therapeutic treatment [5] [6]. In >95% of human cases, *T. cruzi* infection remains undiagnosed until several years later when chronic evolution of progressive disease results in clinical symptoms associated with cardiac damage. A conclusive diagnosis of *T. cruzi* infection then often requires multiple serological tests, in combination with epidemiological data and clinical symptoms. Unfortunately, after complicated diagnosis, no vaccines or therapies are available to treat the chronically infected individuals.

We have, previously, employed an unbiased computational/bioinformatics approach for screening the *T. cruzi* sequence database and identification of potential vaccine candidates [7]. A strategic analysis of the sequence database led to selection of 71 candidates that were unique to *T. cruzi*. Of these, eight candidates (TcG1, TcG2, TcG3, TcG4, TcG5, TcG6, TcG7, and TcG8) were selected for their ability to induce agglutinating antibody response in mice [7]. Further studies indicated TcG1, TcG2, and TcG4 were maximally immunogenic because these were recognized by IgGs in infected mice and dogs (reviewed in [8]), by CD8^+^ T cells in infected mice [9], and elicited type I cytokines (e.g. IFN-γ) in mice and dogs [10]. Immunization with candidate antigens as a DNA vaccine provided a degree of protective immunity (TcG2 = TcG4>TcG1) that substantially controlled acute parasitemia after challenge infection in mice and dogs [10], [11]. The delivery of candidate antigens as heterologous prime/boost vaccines further enhanced the protective efficacy, evidenced by >80% control of acute parasitemia and tissue parasite burden and associated inflammation in heart and skeletal tissue [9], [12].

In this study, we investigated the antibody response to the three candidate antigens (TcG1, TcG2, TcG4) in clinically characterized chagasic patients. Our objectives were to evaluate the antigenicity of the candidate antigens in humans and determine their utility in generation of improved diagnostics for screening of *T. cruzi* infection and disease. Our data demonstrate that the candidate antigens are recognized by antibody responses in chagasic patients from two distinct study sites where diverse strains of the circulating parasites were reported. Further, a multiplex assay consisting of the mixture of the three antigens was highly sensitive and specific in diagnosing *T. cruzi* infection in human patients.

## Materials and Methods

### Parasites

*T. cruzi* trypomastigotes (SylvioX10/4, TCI lineage) were maintained and propagated by continuous *in vitro* passage in monolayers of C2C12 cells. Amastigotes were obtained by incubation of the freshly harvested trypomastigotes in RPMI-10% FBS medium, pH 5.0 at 37°C, 5% CO_2_ for 2 h.

### Human subjects

Human sera samples used in this study were obtained from Salta Argentina (located at the border of Bolivia) and Chiapas Mexico (located on the border of Guatemala) that are known to be endemic for *T. cruzi* transmission and human infection. All procedures were approved by the Institutional Review Boards at the University of Texas Medical Branch (UTMB), Universidad Nacional de Salta (UNSa), Argentina, and the Universidad Autónoma de Chiapas, Mexico. All sera samples obtained for the study were analyzed for *T. cruzi-*specific antibodies by commercially available kits. Those positive by ≥ two tests were considered seropositive (+ve) and those negative for these tests were considered as seronegative (−ve). All samples were decoded and de-identified before they were provided for research purposes. Written informed consent was obtained from all individuals.

In Argentina, all individuals were clinically characterized, and venous blood samples were collected to obtain plasma or serum. Clinical data included medical history, physical examination and subjective complaint of frequency and severity of exertional dyspnea, electrocardiography (12-lead at rest and 3-lead with exercise) to reveal cardiac rhythm and conduction abnormalities and transthoracic echocardiogram to obtain objective information regarding the left ventricular (LV) contractile function. Chest X-ray was used to assess cardiomegaly (cardio-thoracic ratio>0.5). Seronegative and physically healthy subjects exhibiting no history or clinical symptoms of heart disease were used as controls. Seropositive chagasic patients were classified according to clinical exam as follows: Group 0: no echocardiography abnormalities, no left ventricular dilatations, and ≥70% ejection fraction (EF) indicating preserved systolic function, Group I: negligible to minor EKG alterations, EF: 55–70%, no indication of heart involvement; Group II: a degree of heart involvement with systolic dysfunction (EF: 40–55%); and Group III: moderate to severe systolic dysfunction (EF≤40%), left ventricular dilatation (diastolic diameter ≥57 mm), and/or potential signs of congestive heart failure.

In Mexico, human sera samples were collected within the framework of a research project on emerging zoonotic diseases conducted jointly by several institutions, including Chiapas State University (UNACH), Mexican Social Security Institute (IMSS), Chiapas Health Institute (ISECH) and UTMB at Galveston [13]. The seropositive subjects generally represented indeterminate/asymptomatic form of disease. Patients' detailed information is presented in Table 1.

**Table 1:** Characterization of the subjects included for screening of TcG1-,TcG2-, and TcG4-specific antibody responses in this study.

Clinical characterization	Enrolled subjects (numbers)	Age range in years	Sex Males (%)
**Salta, Argentina** a			
*Seropositive for T. cruzi-specific antibodies* b			
Chagasic 0	n = 54	19–75	44 (40%)
Chagasic I	n = 30		
Chagasic II	n = 17		
Chagasic III	n = 9		
*Seronegative for T. cruzi-specific antibodies*			
Healthy, no disease	n = 40	18–55	19 (48%)
Leishmaniasis	n = 35	18–62	25 (71%)
Autoimmune diseases	n = 15	NA	NA
**Chiapas, Mexico** c			
*Seropositive for T. cruzi-specific antibodies*			
Chagasic 0-I	n = 65	18–73	29 (45%)
*Seronegative for T. cruzi-specific antibodies*			
Healthy, no disease	n = 34	18–73	15 (45%)
**Non-endemic areas** d			
Seronegative, healthy, no disease	n = 42	18–55	16 (38%)
Seronegative, other Cardiomyopathy	n = 20	42–75	12 (60%)

a Subjects in Argentina were screened for *T. cruzi*-specific antibodies by Wiener Chagatest-ELISA and Wiener Chagatest-HAI kits. Clinical exam included physical exam, electrocardiography and echocardiography. Confirmation of leishmaniasis was obtained by parasitological test, Montenegro reaction, clinical demonstration, and two PCR approaches. One of the seropositive patient presenting acute infection was referred for chemotherapeutic treatment with Benznidazole.

b Five of the seropositive/chagasic subjects from Argentina were positive for Leishmania infection, determined by two PCR approaches.

c Sera samples from Chiapas Mexico were screened by epimastigote antigenic lysate-based ELISA, trypomastigote-based flow cytometry, and Chagas Stat-Pak immuno-chromatograpic test.

d Seronegative subjects from non-endemic areas were screened for *T. cruzi*-specific antibody response using *T. cruzi* trypomastigote lysate as antigen source in ELISA assays. Seronegative/cardiomyopathy patients were identified based upon blood levels of NT-proBNP to be >2000 pg/ml (normal<450 pg/ml).

N/A: not available.

### 1^st^-phase screening

All sera samples from Salta Argentina were analyzed for *T. cruzi-*specific antibodies by the personnel of the Clinical laboratories at the San Bernardo Hospital using a Wiener Chagatest-ELISA recombinant v.4.0 kit (cut-off absorbance at 450 nm: average of seronegative samples (<0.1) +0.2., i.e. ≥0.3). Serological tests were also done following the specifications of the commercial IHA test kit (Wiener Chagatest-HAI, positive ≥1∶16 dilution). Those positive by both tests were considered seropositive, and exposed to *T. cruzi* infection.

All sera samples from Chiapas Mexico were screened by epimastigote antigenic lysate-based ELISA, trypomastigote-based flow cytometry, and Chagas Stat-Pak immuno-chromatograpic test (Chembio Diagnostic Systems, Medford NY). Those positive by at least two tests were considered seropositive [13].

### 2^nd^-phase screening

Based upon the above analysis, we created sera pools for 2^nd^ phase screening. Briefly, from Chiapas Mexico, a seropositive pool (n = 65) and a seronegative pool (n = 34) was included in the 2^nd^ phase. From Salta Argentina, sera and plasma samples collected in year 2009 (seropositive, n = 65; seronegative, n = 20) and year 2010 (seropositive, n = 45; seronegative, n = 20) were included. All blood samples positive for *T. cruzi-specific* antibodies from Salta Argentina were analyzed for *Leishmania* infection by two PCR approaches. Briefly, total DNA was extracted from blood samples with a phenol-chloroform mixture, precipitated with ethanol and dissolved in 20 µl of TE buffer. A PCR reaction was performed using 1 µl isolated DNA with primer pairs (5′-GTGGGGGAGGGGCGT-TCT-3′ and 5′-ATTTTACACCAACCCCCAGTT-3) that specifically amplify 120 base pair product from the conserved region of *Leishmania* kDNA [14]. The polymorphism-specific PCR (PS-PCR) allows the identification of *Leishmania* species from Argentina, and was performed using the primer pairs as previously described [15].

To evaluate the specificity of the TcG1-, TcG2-, and TcG4-based diagnostic ELISA, sera samples from volunteer donors from Galveston TX, Buenos Aires Argentina, and Toluca Mexico with no history of residence in the endemic areas, and were healthy (n = 42, true negative controls) or exhibited cardiomyopathy of other etiologies (n = 20) were included in the study. Seronegative/cardiomyopathy patients were identified based clinical exam, and blood levels of NT-proBNP to be >2000 pg/ml (normal<450 pg/ml). To examine the cross-reactivity of antigen-based assay, sera samples from subjects living in Salta Argentina, and diagnosed for *Leishmania* infection (n = 35) or certain autoimmune diseases (n = 15) were also included in the study. Patients were diagnosed for leishmaniasis (cutaneous, mucocutaneous or visceral) based upon three criteria (smears and lesions, Montenegro reaction, epidemiology and clinical demonstration) as described in [15], and further identified to be positive for *Leishmania* infection by kDNA-specific PCR and PS-PCR approaches, as above. Briefly, parasitological analysis was done on smears of dermal scrapings stained with May-Grunewald Giemsa and examined under a microscope. For Montenegro skin test, promastigote protein lysates (4 µg/100 µl) of *Leishmania braziliensis* were injected intradermally on the ventral forearm of the patients, and indurations ≥5 mm in diameter, observed 48 h after the injections were considered reactive. Clinical features included the presence of compatible tegumentary injuries with ulcerative, nodulous, papulous cutaneous or mucocutaneous lesions and a congruent epidemiological history. Patients' information is presented in Table 1.

Sera samples were analyzed for IgG antibody levels by using TcG1-, TcG2-, TcG4-based ELISA. Recombinant TcG1, TcG2 and TcG4 proteins were purified from *E. coli*. The nucleotide sequences of TcG1, TcG2 and TcG4 antigens have previously been submitted to GenBank under accession numbers AY727914, AY727915 and AY727917, respectively [7]. The cDNAs encoding TcG1 (1–166 amino acids), TcG2 (1–220 amino acids), and TcG4 (1–92 amino acids) were cloned in pCR2.1 T/A vector, and then sub-cloned in to pET-22b plasmid (Novagen, Gibbstown, NJ) such that the encoded proteins were in-frame with a C-terminal polyhistidine tag. All cloned sequences were confirmed by restriction digestion and sequencing at the Recombinant DNA Core Facility at UTMB. The pET22b plasmids containing TcG1, TcG2 or TcG4 were transformed in *BL21* (DE3) pLysS competent cells (Invitrogen, Carlsbad CA) and recombinant proteins purified using the polyhistidine fusion peptide metal chelation chromatography system (Novagen) [9]. After purification, proteins were dialyzed to remove contaminant particles, and endotoxins, and stored at −80°C till further use. Culture-derived parasites (70% trypomastigotes/30% amastigotes) were lysed by repeated freeze-thaw in PBS (10^9^/ml) and used as a source of *T. cruzi* total antigen for positive control [10].

Flat bottom, high-binding, 96-well plates (Greiner bio-one) were coated overnight at 37°C with 100-µl/well of recombinant antigens (0.5 µg each antigen/well, individually or in combination) or *T. cruzi* total lysate (5×10^5^ parasite equivalents/well). Plates were blocked for 2 h at 37°C with 200-µl/well of 5% non-fat dry milk (NFDM) in PBS, washed with PBS-0.05%Tween-20 (PBST) twice, and PBS once, and then incubated for 2 h with test sera (100-µl/well) added in triplicate in 2-fold dilutions. Plates were then washed and incubated at room temperature for 30 min with 100-µl/well of horseradish peroxidase-labeled goat anti-human IgG (1∶5000 dilution in PBST-1% NFDM), and color was developed for 5 min with 100 µl/well of Sure Blue TMB substrate (Kirkegaard & Perry Laboratories). The colorimetric reaction was stopped with 2N H_2_SO_4_, and absorbance measured at 450 nm using SpectraMax M5 Microplate Reader (Molecular Devices).

The sensitivity of the antigen-based ELISA (2^nd^ phase) was determined by calculating the percentage of chagasic samples that exhibited reactivity with recombinant proteins out of the total samples previously categorized as seropositive based on 1^st^ phase screening with commercially available kits. The data from antigen-based ELISA analysis of sera samples from healthy individuals, other cardiomyopathy and leshmaniasis patients was utilized to calculate the specificity of the assay as follows: [ number of sera samples analyzed – number of sera samples that exhibited false positive reaction or cross-reactivity with TcG1, TcG2 or TcG4/number of sera samples analyzed] ×100.

### Data analysis

All samples were analyzed in duplicate and assayed at least twice for all experiments. Box plots and dot plots were made using Sigma Plot 12.0 and GraphPad Prism 5 software, respectively, and statistical analysis was conducted using SPSS v.18 software. Results were analyzed using Student's t test for statistical evaluation of mean values for experimental and control samples, and the level of significance was taken at α<0.05. Pearson's correlation analysis was performed to determine the relationship between predictive efficacies of the antibody response to selected antigens in diagnosis of disease severity. Seropositivity rates for anti-*T. cruzi* antibodies in different tests, and their confidence intervals [CIs], were calculated using the mid-*P* 95% confidence interval (95% CI) using Epi Info (version 6.0) software.

## Results

To proceed with sample analysis, we optimized ELISA components by cross-titration, using a pool of known positive and negative controls (1∶20–1∶1600 dilutions). The optimal sera and HRP-conjugated secondary antibody dilutions providing maximum signal-to-noise ratio were determined to be 1∶50 and 1∶5,000, respectively, and used in all further investigations. The variations in reactivity of negative and positive sera among different assays and plates of the same experiment ranged from 3–12%.

**Figure 1:**
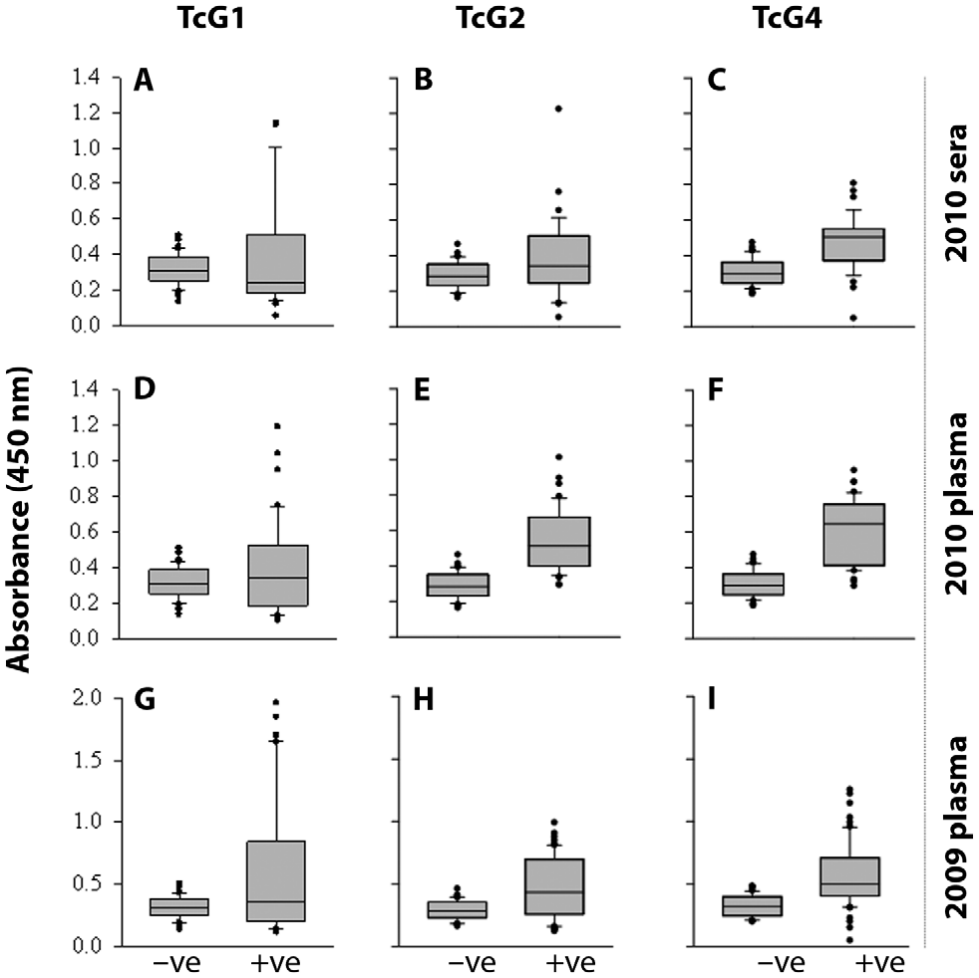
Antigenicity of TcG1, TcG2 and TcG4 in inhabitants of Salta Argentina. Sera (A–C) and plasma (D–I) samples obtained in year 2010 (A–F) and year 2009 (G–I) from volunteers in Salta Argentina were identified as seropositive (+ve) or seronegative (−ve) in 1^st^-phase screening by using conventional approaches. The 2^nd^-phase screening for antigen-specific antibody response was conducted by an ELISA using the recombinant TcG1, TcG2 and TcG4 proteins. Data (mean of four observations from each sample) are presented as box plot. The horizontal lines of the box (bottom to top) depict the lower quartile (Q1, cuts off lowest 25% of the data), median (Q2, middle value), and upper quartile (Q3, cuts off the highest 25% of the data). The lower and upper whiskers depict the smallest and largest non-outlier observations, respectively, and solid dots represent the outliers. The spacing between the different parts of the box indicates the degree of dispersion (spread). Standard deviation for all samples was <12%.

We, first, monitored the antigenicity of TcG1, TcG2, and TcG4 using sera samples collected from volunteers enrolled in the study in Argentina in year 2010. Samples were stored at −80°C just after collection, and thawed when utilized. The negative sera samples (n = 20) from the endemic area near Argentina-Bolivia border exhibited low reactivity for TcG1, TcG2, and TcG4, similar to that noted for confirmed negative controls (n = 42) from non-endemic areas (TcG1: 0.216±0.035 versus 0.211±0.048, TcG2: 0.240±0.04 versus 0.230±0.044, TcG4: 0.225±0.041 versus 0.252±0.038, expressed as mean absorbance ± SD). In comparison, a 4-fold, 2.75-fold, and 2.65-fold increase in sera levels of antibody response to TcG1, TcG2, and TcG4, respectively, was noted in previously characterized seropositive subjects (n = 45) from Argentina-Bolivia border (TcG1: 0.81±0.33, TcG2: 0.66±0.20, TcG4: 0.57±0.09, expressed as mean absorbance ± SD, p<0.001 for all, Fig. 1.A–C). The sera levels of antibodies to TcG1, TcG2, and TcG4 were above the mean_seronegative_ level in 62.2%, 66.6% and 75.5% of the 1^st^-phase seropositive subjects. When analyzing plasma samples from the same individuals, we noted a 3.34-fold, 2.4-fold and 2.3-fold increase in plasma levels of antibodies to TcG1, TcG2 and TcG4 in seropositive subjects as compared to seronegative controls (TcG1: 0.77±0.21 versus 0.20±0.05, TcG2: 0.65±0.12 versus 0.21±0.057, TcG4: 0.67±0.09 versus 0.20±0.048, expressed as mean absorbance ± SD, p<0.001 for all, Fig. 1.D–F). The plasma levels of antibodies to TcG1, TcG2, and TcG4 were above the mean_seronegative_ levels in 71.1%, 77.7% and 80% of the 1^st^-phase seropositive subjects. These data suggested that a) TcG1, TcG2 and TcG4 are recognized by antibody responses elicited in human patients infected by *T. cruzi*, and b) both plasma and sera samples can be utilized to monitor the antibody response.

We then analyzed the plasma samples that were collected in 2009, and characterized as seropositive (n = 65) and seronegative (n = 20) by 1^st^-phase serology tests. These samples were subjected to two cycles of freezing/thawing during the two-year storage. Our data showed a 6.72-fold, 2.4-fold and 2.9-fold increase in plasma levels of antibodies to TcG1, TcG2 and TcG4 in seropositive subjects as compared to seronegative controls (TcG1: 1.66±0.55 versus 0.204±0.05, TcG2: 0.69±0.13 versus 0.239±0.039, TcG4: 0.75±0.20 versus 0.227±0.05, expressed as mean absorbance ± SD, p<0.001 for all, Fig. 1.G–I). The plasma levels of antibodies to TcG1, TcG2, and TcG4 were above the mean_seronegative_ level in 61.5%, 64.6% and 81.5% of the seropositive subjects. These results suggest that antibody response to TcG1, TcG2, and TcG4 is stable, and field samples can be utilized to examine antigenicity of the selected candidates in large-scale population studies. Overall, the data presented in Fig. 1 also indicate that TcG1, TcG2 and TcG4 are expressed by *T. cruzi* isolates circulating in the endemic areas at the Argentina-Bolivia border.

**Figure 2:**
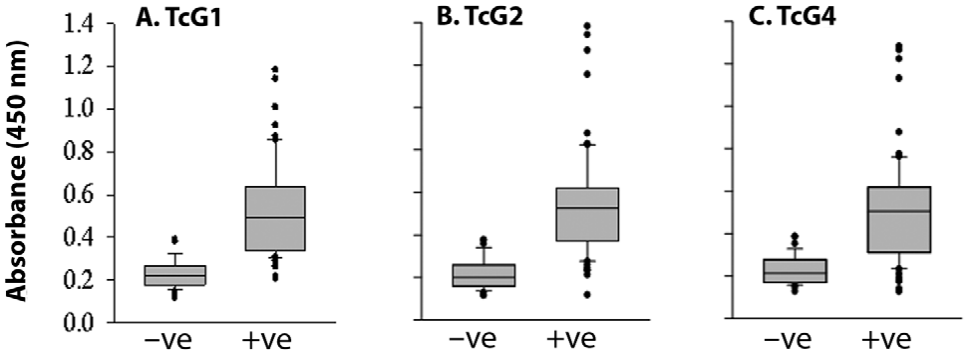
TcG1, TcG2 and TcG4 are recognized by antibody response in human subjects from Mexico. Sera samples obtained from volunteers living in the endemic areas of Chiapas Mexico were characterized as seropositive (+ve) and seronegative (−ve) by whole-parasite antigen based serology tests in the 1^st^ phase. The TcG1 (A), TcG2 (B) and TcG4 (C) specific antibody response was measured by ELISA, and data are presented as box plot (details in Fig. 1).

It is important to know if antibody recognition of the three antigens can be expanded for diagnosis of *T. cruzi* infection in other countries where different isolates are suggested to be present in domestic and sylvatic cycle of parasite circulation. For example, in Argentina and neighboring countries in South America, TCII isolates are predominantly identified in peripheral blood of seropositive patients, though molecular studies have revealed the presence of TCI parasites also in heart biopsies of chronic chagasic patients [16], [17], [18]. In Mexico and Guatemala, TCI is dominantly found in epidemiological evaluation of infected triatomines as well as in blood samples from acute and chronic chagasic cardiomyopathy patients [19], [20]. We, therefore, monitored the antigenicity of TcG1, TcG2, and TcG4 in human sera samples from Mexico-Guatemala border area that were characterized as seropositive (n = 65) and seronegative (n = 34) by 1^st^-phase serology tests. Our data showed 6.72-fold, 2.4-fold and 2.9-fold increase in sera levels of antibodies to TcG1, TcG2 and TcG4 in seropositive samples as compared to that noted in seronegative healthy controls (TcG1: 0.7±0.25 versus 0.211±0.047, TcG2: 0.64±0.22 versus 0.25±0.05, TcG4: 0.65±0.20 versus 0.212±0.048, expressed as mean absorbance ± SD, p<0.001 for all, seropositive versus seronegative, Fig. 2). The sera levels of antibodies to TcG1, TcG2, and TcG4 were above the mean_seronegative_ level in 69.2%, 76.9% and 72.3% of the seropositive subjects from Mexico. These data demonstrate that TcG1, TcG2 and TcG4 are antigenic, and recognized by antibody responses in chagasic patients from Mexico, and suggest that the three antigens are expressed by *T. cruzi* isolates circulating in Mexico-Guatemala border area.

**Figure 3:**
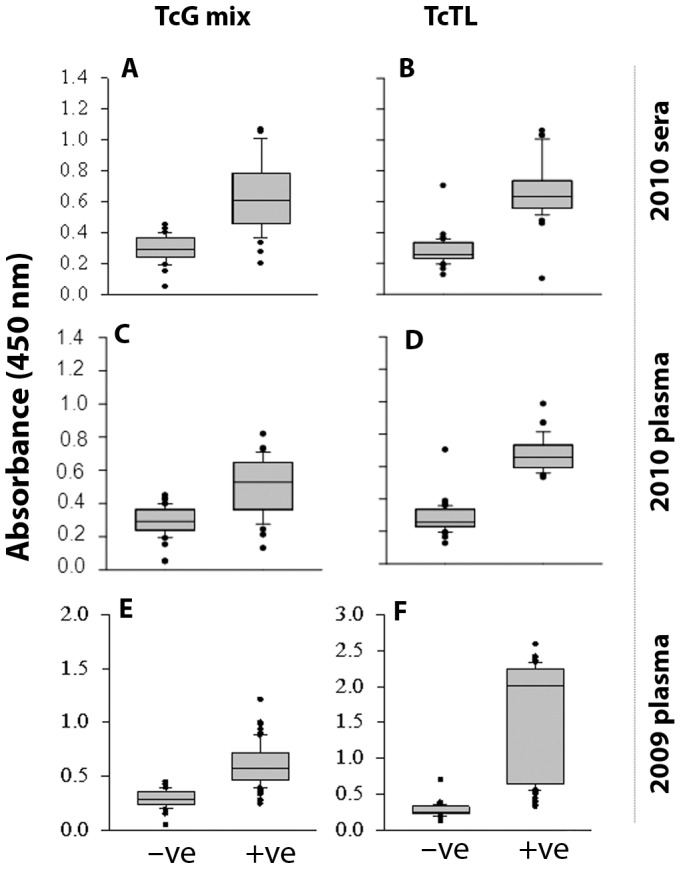
TcG_mix_-based ELISA provides superior efficacy in identifying exposure to *T. cruzi* infection among inhabitants of Salta Argentina. Sera (A&B) and plasma (C–F) samples, characterized as seropositive (+ve) and seronegative (−ve) by conventional approaches, were submitted to multiplex ELISA using the mixture of recombinant TcG1, TcG2 and TcG4 proteins for capturing the antigen-specific antibody response (A,C,E). Shown are the antibody response captured using the *T. cruzi* trypomastigote lysate (TcTL) as a source of crude antigen (B,D,F) for comparison purpose.

Next, we determined if the three candidate antigens can be utilized together to improve the diagnosis of exposure to *T. cruzi*. For this, we coated the 96-well plates with either the mixture of TcG1, TcG2 and TcG4 (0.5 µg/well each) or *T. cruzi* trypomastigote lysate (TcTL, 2×10^5^ parasite equivalent), and monitored the antibody response by ELISA under similar experimental conditions. When antibody response was captured using the TcG_mix_, the seronegative controls from the non-endemic areas exhibited a mean absorbance ± SD of 0.225±0.039. Using the controls' mean absorbance+2SD, our data validated 40 of the 45 sera samples (88.8%) from Argentina, characterized as seropositive in 1^st^-phase screening in the year 2010, were seropositive for TcG_mix_-specific antibodies (mean absorbance ± SD: 0.73±0.17, maximum OD: 1.2, Fig. 3A). One of the volunteer previously characterized as seronegative exhibited anti-TcG_mix_ antibody response above the mean_seronegative_ level. Similarly, the plasma detection of antibody response to TcG_mix_ identified 102/110 of the seropositive subjects (92.7%) identified in 1^st^-phase screening in the years 2009 and 2010 (Fig. 3B,C). In comparison, when plates were coated with TcTL to capture anti-*T. cruzi* antibodies, the seronegative true controls from non-endemic areas, exhibited a mean absorbance ± SD value of 0.233±0.044 (Fig. 3C,E). Using the mean absorbance for controls +2 SD as a cut off, our data validated 97.7–100% sera and plasma (year 2010) and 96.9% plasma (year 2009) samples characterized as seropositive in 1^st^-phase screening in Argentina were also positive for TcTL-specific antibodies; the mean absorbance ± SD for the positive population was 1.1±0.6 (year 2009) and 0.73±0.08 (year 2010) with the highest value being 2.5 and 0.98, respectively (Fig. 3B,D,F). One of the volunteer previously characterized seronegative exhibited anti-TcTL antibody response.

**Figure 4:**
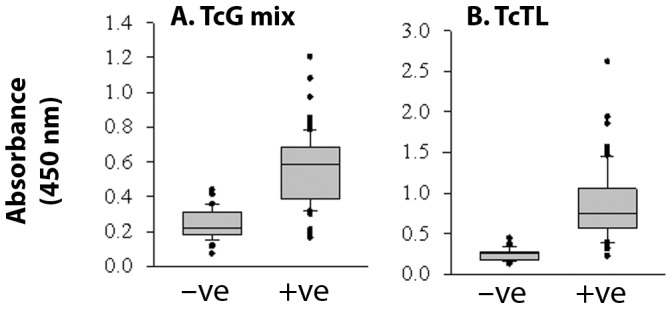
TcG_mix_-based ELISA was effective in diagnosing exposure to *T. cruzi* infection among inhabitants of Chiapas Mexico. Sera samples obtained from volunteers living in the endemic areas of Chiapas Mexico were characterized as seropositive (+ve) and seronegative (−ve) by whole-parasite antigen based serology tests in the 1^st^ phase. Shown are the antibody response captured by an ELISA using mixture (TcG_mix_) of recombinant TcG1, TcG2 and TcG4 proteins (A) or *T. cruzi* trypomastigote lysate (TcTL) (B) as a source of antigen.

To validate that diagnostic potential of the TcG_mix_ based ELISA is not restricted to samples from Argentina, we monitored the antibody response using sera samples collected in Mexico. Of the 65 samples characterized as seropositive in 1^st^-phase screening, 58 (89.2%) and 63 (96.9%) exhibited reactivity when plates were coated with TcG_mix_ and TcTL antigens, respectively (Fig. 4). The mean absorbance ± SD for the antibody response to TcG_mix_ and TcTL in the positive population positive population was 0.75±0.14 (max: 1.2) and 0.87±0.4 (max: 2.6), respectively, the difference between the two values being observed non-significant. No significant difference was observed when either plasma or sera were used as the source of antibodies.

Five of the 110 seropositive/chagasic subjects from Argentina exhibited reactivity for *Leishmaina*-specific antibodies and were likely infected with both pathogens. To examine if the antigen-based ELISA is specific for *T. cruzi* detection, we performed recombinant antigens-specific ELISA using the sera samples from non-chagasic individuals (total 112), including leishmaniasis patients (n = 35), volunteer donors with cardiomyopathy (n = 20) and autoimmune diseases (n = 15) of other etiologies, and healthy donors from non-endemic areas (n = 42), and calculated the specificity of the antigen-based ELISA. Sera samples from leishmaniasis patients (n = 35) exhibited very low reactivity for TcG1, TcG2, and TcG4, similar to that noted for sera samples from confirmed negative controls from non-endemic areas (TcG1: 0.18±0.04 versus 0.21±0.048, TcG2: 0.15±0.06 versus 0.230±0.044, TcG4: 0.232±0.05 versus 0.252±0.038, expressed as mean absorbance ± SD). Likewise, sera samples from patients exhibiting symptoms of cardiomyopathy of non-chagasic etiology or autoimmune diseases showed reactivity to TcG1, TcG2, and TcG4 below the cut-off threshold values derived from healthy/seronegative controls. The specificity for TcG_mix_ was highest (98%), followed by TcG2 (96%), TcG4 (94.6%), TcG1 (93.6%) and TcTL (77.8%), determined by detection of false positive signal for 2, 4, 6, 7 and 25 of the samples, respectively, out of the total 112 samples from non-chagasic individuals that were submitted for antigen- and TcTL-based ELISA. These data indicated that the recombinant antigens were highly specific for the detection of anti-*T.cruzi* antibodies than the whole parasite (trypomastigote) lysate, and exhibited no cross-reactivity to *Leishmania*-specific antibodies.

**Figure 5:**
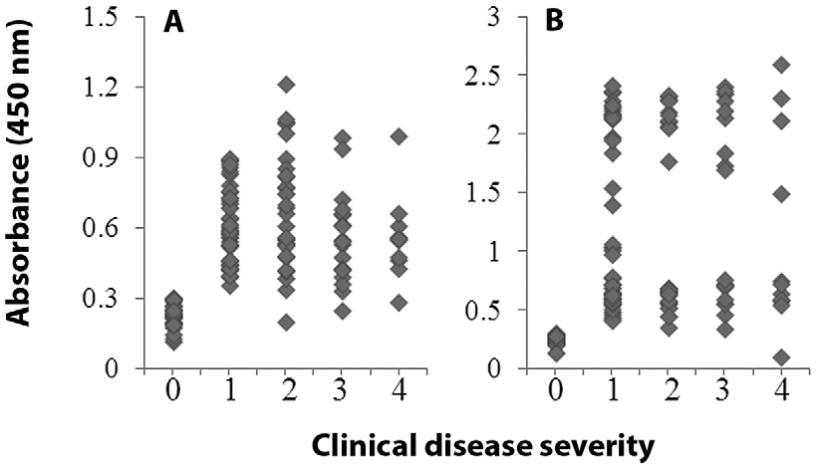
Pair-wise correlation analysis. Shown is pair-wise correlation analysis of antibody response to TcG_mix_ (A) and TcTL (B) with clinical disease category in patients enrolled in the study from Argentina-Bolivia border area. For this, seronegative/healthy subjects were labeled as 0, and patients classified as 0, I, II and III (Materials and Methods) were labeled as 1, 2, 3, and 4, respectively. Dots, individual subjects.

Pearson correlation analysis was employed to identify correlation between antigen-specific antibody response and disease state including the data derived from seronegative/healthy controls and seropositive/chagasic subjects. For this, seronegative/healthy subjects were labeled as 0, and patients classified as 0, I, II and III (Materials and Methods) were labeled as 1, 2, 3, and 4, respectively. Antibody response was titrated using 2-fold sera dilutions (1∶50–1∶1600). We observed no significant correlation between the anti-TcG2, anti-TcG4, anti-TcG_mix_ and anti-TcTL antibody titration curves and clinical disease category in any of the patient population (data not shown). The representative correlation data from sera levels of anti-TcG_mix_ and anti-TcTL antibody response (1∶50 dilutions) and clinical disease category for the clinically characterized Argentine patients enrolled in the study is shown in Fig. 5. It is worth noting that TcG_mix_-specific antibodies exhibited a clear downward trend with patients' disease severity, indicating that presence of antibodies for TcG1, TcG2, and TcG4 is protective during progressive Chagas disease (Fig. 5A). No clear trend or correlation was observed for TcTL specific antibody response and disease severity in any of the patient population (Fig. 5B).

## Discussion

Serological diagnosis of Chagas disease is frequently based on tests such as enzyme-linked immunoassays (EIAs), indirect immunofluorescence assays, and indirect haemagglutination assays (IHAs). Most of these serological tests, including one recently licensed by the United States Food and Drug Administration for use as a blood screening test in the U.S. [21], use crude or semi-purified *T. cruzi* epimastigote forms as the antigen, the stage that is present in insects but not in infected humans. Overall, the current diagnostics fail to provide a high degree of sensitivity and specificity, requiring use of multiple tests for diagnosis of *T. cruzi* infection [22]. Further, most of the currently available kits produce questionable results when used for donors with low titers [23]. The absence of a true gold standard makes it difficult for the medical practitioners to provide proper treatment as not all cases are properly identified and treatment response cannot be accurately monitored. Accordingly, World Health Organization has emphasized the need to employ defined antigens for improved serodiagnosis of *T. cruzi* infection [24]. However, before an antigen can be used for diagnostics, several criteria should be met: (i) the selected candidate antigen(s) should be expressed in isolates circulating in different areas of endemicity, (ii) antigens should be highly immunogenic in populations with different genetic background, (iii) they should be absent from other pathogens to prevent cross-reactivity, and (iv) easily expressed and purified from traditional protein expression systems (e.g. *E. coli*) to ensure reproducibility and quality control [25]. Because of the complexity involved in antigen selection and testing, limited progress has been made towards the development of antigen-based kits for the diagnosis of *T. cruzi* infection.

Keeping the above guidelines in mind, we believe that TcG1, TcG2 and TcG4 are ideal candidates for the development of diagnostic assays. One, these antigens were previously shown to be expressed in infective and intracellular forms of clinically relevant multiple isolates of *T. cruzi* [7]. Two, the small size of the three antigens (TcG1: 18.4 kDa, TcG2: 24 kDa, TcG4: 10 kDa) allows reproducible high-yield purification of the proteins from *E. coli* (0.5–1.0 g/L) and is amenable to large-scale production. Three, the presence of multiple 12-mer B cell epitopes of high specificity (Table 2) suggests that these antigens will be immunogenic, recognized by B cells of the immune system, and elicit antibody response. Indeed, we have found that TcG1, TcG2 and TcG4 are recognized by antibodies elicited in infected dogs and mice [9], [11]. The diagnostic potential of the three antigens in humans was evident from our analysis of a panel of sera or plasma from chronic chagasic patients from Argentina-Bolivia and Mexico-Guatemala border areas. *T. cruzi* strains of lineages TCV (IId), TCII (IIb) and TCI were identified in seropositive chagasic patients from Argentina/Bolivia (Patricio Diosque and Monge Rumi, personal communication), and of lineage TCI in seropositive subjects from Mexico/Guatemala (unpublished results). Others have documented the predominance of TCII isolates in peripheral blood of seropositive patients from South America [16], [17], [18] and TCI in Mexico and Guatemala [19], [20]. Thus, our data presented in Figs.1, 2, 3, 4 suggest the diagnostic kit developed using TcG1, TcG2 and TcG4 will be useful in identifying *T. cruzi* circulation and transmission in South, Central and North America.

**Table 2:** B cell epitopes in candidate antigens.

Protein name (Acc# in Genbank)	Protein (aas)	Predicted B cell epitopes		
		Position	Epitope	Score
TcG1 (AAU47265.1)	166	14	RIIRGPRQDRVG	1
		154	VDSKPAAKKRIS	0.947
		66	SRNCSTRTLKNV	0.904
		127	VFDENDQKKPVS	0.788
		109	ERYQLRVAKRSR	0.699
		27	VVDIIDGNRVLV	0.667
TcG2 (AAU47266.1)	221	113	FYATDGNAANYT	0.99
		144	EKEKTSTNRRSK	0.838
		161	YDISGSNTNLCD	0.815
		127	AAVDGGVAHRSL	0.793
		7	ESGFVPSDGMRR	0.751
		190	SVHDSKDVSPQK	0.716
		210	EAFRIRLPPLLG	0.715
		91	PKHFVAPLNSNS	0.667
TcG4 (AAU47268.1)	91	39	MWVEHQRRLRQE	0.973
		1	MSAKAPPKTLHQ	0.958
		74	IPTIVPKELHEL	0.542

Linear B cell epitopes (12 amino acid lengths) were predicted using a BCPred tool (http://ailab.cs.iastate.edu/bcpreds/).

It is important to note that TcG1, TcG2 and TcG4, when used individually, exhibited 93.6–96% specificity in detecting anti-*T. cruzi* antibody response that was in the range for other recombinant proteins (e.g. TSSA, CP1 and CP2) proposed for Chagas disease immunodiagnosis [26], [27]. However, 98% specificity of the TcG_mix_-based ELISA was much higher than that observed for single antigens in this study or other recombinant antigens in other reports [26], [28]. Further, TcG_mix_ specificity at 98% was significantly better than that observed with *T. cruzi* trypomastigote-based ELISA (77.8%) that is not desirable because culturing of human-infective form of the pathogen requires special facilities, technology, and expertise.

Another major concern that is generally not taken into consideration when one is using serological tests for Chagas disease is the potential frequency of cross-reactivity. In some areas of endemicity in Central America and Brazil, where *T. cruzi* and the nonpathogenic protozoan *Trypanosoma rangeli* can be found infecting the same vectors and vertebrate hosts, cross-reactivity has been proposed to contribute to miscalculated higher percentage of *T. cruzi* transmission and misdiagnosis of patients may have severe health-related and economic consequences [29], [30]. Others have documented the crude antigen-based serodiagnostic kits exhibit cross-reactivity between sera of patients infected with *T. cruzi* and sera of patients infected with *Leishmania* spp. [31], [32], [33]. In our study, homology searches suggested that only TcG1 is similar to a ribosomal protein of other trypanosomes (>90% identical) while TcG2 and TcG4 exhibited no clear paralog in the public databases (Fig.S1). Thus, to rule out the possibility of cross-reactivity with *Leishmania*, we have screened sera samples from confirmed leishmaniasis patients for antibody cross-reactivity to TcG1, TcG2 and TcG4. Our data clearly showed that the three antigens exhibited no cross-reactivity to antibodies generated in cutaneous, mucocutaneous and visceral leishmaniasis patients, providing evidence that the antibody response to TcG1, TcG2 and TcG4 was solely directed by exposure to *T. cruzi* infection.

Finally, TcG_mix_-ELISA performed at a higher potency in discriminating weakly positive samples from background, demonstrated statistically by calculating the quotient of measured optical density values divided by the cutoff values. Only 12 of all the seropositive samples had a quotient smaller than 2.0 in the TcG_mix_-ELISA whereas TcG1-, TcG2- TcG4 and TcTL-based ELISA resulted in 18, 16, 14 and 16 samples below the mean value. This potency will facilitate diagnosis because weakly positive results routinely need to be confirmed by alternative assays. Thus, based upon high sensitivity (93%) and specificity (98%) to sera from chagasic patients from different endemic countries, we surmise that TcG_mix_-based ELISA can serve as a single assay to determine the *T. cruzi* status of a given blood sample, and diagnose Chagas disease. We plan to further standardize the assay for large-scale screening and establishing the prototype assay for commercial use of the selected antigens for diagnostic kits.

The immune interactions necessary to eradicate *T. cruzi* parasites are extremely complex and require both humoral and cell-mediated components of the immune system. Previous experimental studies and a few studies in humans indicate that antibodies are able to kill *T. cruzi* in the presence of phagocytic cells, such as macrophages. Others have shown in B cell knockout mice or mice depleted of B cell function, the pivotal role of antibody molecules in attaining resistance to *T. cruzi* infection and Chagas disease (reviewed in [34]). In this study, the mean antibody response to TcG1, TcG2 and TcG4 (individually or in combination) exhibited a downward trend in correlation to clinical disease severity suggesting that antibody response to the candidate antigens was protective, though further studies with larger patient population will be required to validate the significance of this observation. The experimental data indicating the up regulation of IgG1 antibodies specific to TcG1, TcG2 and TcG4 [9], [11] suggest that isotypes related to up regulation of opsonization, cell dependent cytotoxicity and activation of classical complement pathway are elicited by the candidate antigens, and might be present in significant levels in seropositive/chagasic individuals. Considering that the three antigens have been shown to be located on plasma membrane of trypomastigote/amastigote stages of *T. cruzi* by flow cytometry [7], we predict that these will be available as a target to antibody-dependent cell cytotoxicity effector mechanisms mediated by IgG1. Indeed, TcG1, TcG2 and TcG4 have been shown to elicit potent trypanolytic antibodies in accordance with the intensity of the surface expression of these antigens in infective and intracellular stages (as compared to eight others tested in similar experiments) [7]. In addition to IgG1, candidate antigens elicited IgG2b known to drive the type 1 adaptive immunity in experimental mice and dogs [9], [11]. High levels of candidate antigens-induced IgG2b in vaccinated mice were linked to complement-dependent trypanolytic activity [9]. Patients in the indeterminate phase display higher levels of lytic antibodies compared with patients with chagasic heart disease indicating an association between the presence of lytic antibodies and a protective response in chronic patients [35], [36]. This was also evidenced by absence of lytic antibodies in patients treated with anti-parasite drugs that displayed negative hemocultures for over ten years [37]. Based upon these observations, we consider that activation of IgG2a (along with IgG1) will be an important attribute that contributes to host protection against *T. cruzi* infection and Chagas disease when TcG1, TcG2 and TcG4 will be tested as vaccine candidates in humans.

In summary, this is the first report identifying the antigenicity of TcG1, TcG2 and TcG4 in humans. We have concluded that the three candidate antigens are recognized by IgGs in sera samples of seropositive/chagasic patients. Further, the candidate antigens were represented in diverse strains of *T. cruzi* as the sera samples from two different endemic areas from the South, Central and North America recognized the antigens at the same rate. Our data show the serology test developed using the TcG_mix_ is a significantly better alternative to epimastigote extracts currently used in *T. cruzi* serodiagnosis or the trypomastigote lysate used in this study for comparison purposes. TcG_mix_ ELISA was >98% specific and 93% sensitive, was not discriminatory of sex, age or geographic location of the individuals, performed at a higher potency in identifying weakly positive samples, and, thus, has a potential to serve as a single assay for the diagnosis of *T. cruzi* infection. Future studies with large cohorts of patients will be required to determine if immunological responsiveness to the three antigens detects with high confidence the chronicity of infection, severity of disease, and effectiveness of treatment etc.

## Supporting Information

Figure S1 **TcG1, TcG2 and TcG4 homology to proteins in other trypanosomatids.** Homology searches were performed using the NCBI BLASTP against nr database to identify homology for TcG1, TcG2 and TcG4 proteins of *T. cruzi* in other trypanosomatids (e.g. *Leishmania spp, T. brucei, T. congolense, T. vivax*). Shown are the results from maximum observed homology.
(DOC)
