# Supplementary material for: Antigenicity and Diagnostic Potential of Vaccine Candidates in Human Chagas Disease
Source: PLoS Negl Trop Dis. 2013 Jan 17;7(1):e2018. doi: 10.1371/journal.pntd.0002018 (PMC3547861; doi:10.1371/journal.pntd.0002018)
Supplement: Figure S1 — TcG1, TcG2 and TcG4 homology to proteins in other trypanosomatids. Homology searches were performed using the NCBI BLASTP against nr database to identify homology for TcG1, TcG2 and TcG4 proteins of T. cruzi in other trypanosomatids (e.g. Leishmania spp, T. brucei, T. congolense, T. vivax). Shown are the results from maximum observed homology. (DOC) [file pntd.0002018.s001.doc]

**Supplemental Fig.1: TcG1, TcG2 and TcG4 homology to proteins in other trypanosomatids**

**TcG1 versus *Leishmania* spp** [GENE ID: 5415626 LBRM_22_1420](http://www.ncbi.nlm.nih.gov/sites/entrez?db=gene&cmd=search&term=5415626&RID=97X09FPT016&log$=geneexplicitprot&blast_rank=12) | putative 40S ribosomal protein L14 (alternate name Ldp23)

Score = 250 bits (639), Expect = 4e-82, Method: Compositional matrix adjust.

Identities = 116/164 (71%), Positives = 138/164 (84%), Gaps = 0/164 (0%)

TcG1 1 MVKANYIRAGRLVRIIRGPRQDRVGVVVDIIDGNRVLVENPADKKMWRHVQNLKNVEPLK 60

MVK++YIRAGR+VRI+RGPRQDRVGV+VDI+D NRVLVENP D KMWRHVQNLKNVEPLK

L14 1 MVKSHYIRAGRMVRILRGPRQDRVGVIVDIVDANRVLVENPEDAKMWRHVQNLKNVEPLK 60

TcG1 61 FSVELSRNCSTRTLKNVLAEKKILEKYAATKSARRIAAKRAFARSTDFERYQLRVAKRSR 120

+ V + RNCS + LK+ L K+LEKYA T++A R+ AK+A A STDFERYQLRVA+RSR

L14 61 YCVSVGRNCSAKALKDALDSSKVLEKYAKTRTAARVEAKKACAASTDFERYQLRVARRSR 120

TcG1 121 AFWTRKVFDENDQKKPVSWHKVALKKLQKNAKKVDSKPAAKKRI 164

A+W RKVFDE D K PVSWHKVALK++QK A K+DS AK+R+

L14 121 AYWARKVFDEKDAKTPVSWHKVALKRMQKKASKMDSTEGAKRRM 164

**TcG2 versus *Leishmania* spp** [GENE ID: 5419087 LBRM_34_0270](http://www.ncbi.nlm.nih.gov/sites/entrez?db=gene&cmd=search&term=5419087&RID=97WK1PM4014&log$=geneexplicitprot&blast_rank=7) | hypothetical protein

Score = 83.6 bits (205), Expect = 5e-16, Method: Compositional matrix adjust.

Identities = 58/155 (37%), Positives = 72/155 (46%), Gaps = 30/155 (19%)

TcG2 1 MSLSFIESGFVPSDGMRR--GVEAADTSAAAELLHLAVPPLMDAGGKTRVCVAFYEAAQC 58

MS S FVPS GV+ T AA K VC+AFYE +C

LmHp 1 MSFSIHTRAFVPSPAAYSPSGVKEKTTEAA--------------HAKDCVCIAFYENGKC 46

TcG2 59 PFDSRCEHAHHFSELNGYTQNKLLETVPVESIPKHFVAPLNSNSSSGNNKNDRTFYATDG 118

P+DS+CEHAHHFSEL+ TQ +LL+ V V SIP HF + +D+ D

LmHp 47 PYDSQCEHAHHFSELSIETQTRLLQCVSVSSIPPHFF--------DASQPHDKMLTTLDA 98

TcG2 119 NAANYTATAAVDGGVAHRSLGGEHGEKEKTSTNRR 153

A T AA D R + GE E+T + RR

LmHp 99 -ALPKTVMAAYD-----RYSIIQRGEAERTVSMRR 127

**TcG4 versus *Leishmania* spp** ([GENE ID: 5069524 LINJ_25_0600](http://www.ncbi.nlm.nih.gov/sites/entrez?db=gene&cmd=search&term=5069524&RID=97W5MYTT016&log$=geneexplicitprot&blast_rank=4) | hypothetical protein

Score = 106 bits (265), Expect = 8e-28, Method: Compositional matrix adjust.

Identities = 49/90 (54%), Positives = 65/90 (72%), Gaps = 0/90 (0%)

TcG4 2 SAKAPPKTLHQVRNVAYIFAAWAGLQKGFAEKSANDKMWVEHQRRLRQENAKRQHAAHAL 61

+AK PKTL+Q RN +Y+ AW G KGF EK+AND WV HQ+R+RQ+N +R AA A+

LmHp 7 AAKVAPKTLNQFRNFSYLVVAWLGFNKGFREKAANDAEWVAHQQRVRQQNVERHQAAQAM 66

TcG4 62 EELKQDEELERSIPTIVPKELHELVKALEK 91

E KQ+ ELE +IP +VP+ LHE+ K +EK

LmHp 67 AEAKQNAELETTIPAMVPEGLHEVYKDVEK 96

Homology searches were performed using the NCBI BLASTP against nr database to identify homology for proteins in other trypanosomatids (e.g. *Leishmania spp, T. brucei, T. congolense, T. vivax*). Shown are the results from maximum observed homology.
